# The Neonatal QRS Complex and Its Association with Left Ventricular Mass

**DOI:** 10.1007/s00246-023-03361-0

**Published:** 2023-12-27

**Authors:** Julie Molin, Joachim Hartmann, Maria Munk Pærregaard, Caroline Boye Thygesen, Anne-Sophie Sillesen, Anna Axelsson Raja, Ruth Ottilia Birgitta Vøgg, Kasper Karmark Iversen, Henning Bundgaard, Alex Hørby Christensen

**Affiliations:** 1 Department of Cardiology, Herlev-Gentofte Hospital, Copenhagen University Hospital, Copenhagen, Denmark; 2 The Capital Regions Unit for Inherited Cardiac Diseases, Department of Cardiology, The Heart Center, Rigshospitalet, Copenhagen University Hospital, Copenhagen, Denmark; 3 Department of Clinical Medicine, University of Copenhagen, Copenhagen, Denmark

**Keywords:** Electrocardiography, Neonates, QRS complex, Left ventricular mass, Reference values

## Abstract

To evaluate QRS complex features during the first month of life and the association with echocardiographic measurements of left ventricular mass in neonates. Prospective cohort study of neonates with electrocardiography (ECG) and echocardiography performed during the first month of life. Left ventricular mass index (LVMI) was determined by echocardiography and the correlation with electrocardiographic markers of LVMI outliers (≥ 98th percentile) were analyzed. We included 17,450 neonates (52% boys; median age at examination 11 days) and found an increase in median QRS duration and LVMI during the first month of life (54 vs. 56 ms and 24.7 vs. 28.6 g/m^2^ at days 0–4 and 25–30, respectively; both *p* < 0.001). All investigated ECG features (QRS duration, QRS area in V1/V6, maximum amplitudes of S-V1/R-V6, and the Sokolow–Lyon voltage product) showed no to low correlation with LVMI, resulting in low sensitivities (0–9.0%), but high specificities (97.2–98.1%), and area under the curve values close to the identity line (0.49–0.61) for identifying LVMI outliers. Adjustment of outlier definition for LVMI and threshold for QRS features had no significant effect on sensitivity. We present reference values for QRS complex features and their association with LVMI in neonates from a large, unselected, population-based cohort. The QRS complex gradually evolved during the first month of life but had a low correlation with LVMI. Our results indicate a poor diagnostic value of using ECG features to identify LVMI outliers in neonates.

*Trial Registry* Copenhagen Baby Heart, NCT02753348, https://clinicaltri-als.gov/ct2/show/NCT02753348?cond=Copenhagen+Baby+Heart&draw=2&rank=1, deidentified individual participant data will not be made available.

**Supplementary Information:**

The online version contains supplementary material available at 10.1007/s00246-023-03361-0.

## Introduction

The QRS complex reflects the depolarisation of the ventricular myocardium and is an important diagnostic and prognostic parameter in many clinical settings [1–3]. The duration and amplitudes of the QRS complex may be markers of altered conduction or structural/functional abnormalities of the left ventricle, including left ventricular hypertrophy (LVH) or dilatation [1]. The electrocardiogram (ECG) is a diagnostic cornerstone in cardiology and a valuable tool in screening for numerous cardiac diseases, including LVH in adults [4], and has predictive power for cardiovascular outcomes such as stroke, myocardial infarction, and death [5–8].

Numerous processes may lead to LVH, including adaptation to increased hemodynamic stress [9], alterations secondary to congenital heart disease (CHD), maternal diabetes mellitus [10], metabolic- and neuromuscular diseases, as well as hypertrophic cardiomyopathy [11]. Hypertrophic cardiomyopathy has been found to be one of the most common causes of sudden cardiac death in young people [11] underlining the importance of an early diagnosis of LVH. Echocardiography is a non–invasive tool useful for determining left ventricular structure/function and can reliably detect LVH in adults [12]. However, the ECG is widely available and ECG-LVH is an established prognostic marker [13]. Previous studies have investigated the usefulness of standard ECG for detecting LVH in children and have found conflicting results [9, 14–17]. Several ECG features including QRS area, maximum precordial amplitudes, voltage product, etc., have been investigated in relation to left ventricular mass (LVM) with variable sensitivity and specificity. However, previous studies were performed in smaller, selected cohorts, with a large variation in age (0–18 years) spectrums [9, 15].

Definite evidence on whether ECG features are reliable predictors of left ventricular mass index (LVMI) outliers in neonates requires data from a large, population-based cohort with systematic, concurrent electro- and echocardiographic evaluation. We assessed QRS complex features during the first month of life and investigated the association between electrocardiographic features and echocardiographic measurements of LVMI in a large cohort of asymptomatic, consecutively enrolled neonates.

## Methods

### Study Design

The Copenhagen Baby Heart Study is a prospective, multicentre, population-based cohort study of neonatal cardiac structure and function. All expectant parents in the three major maternity units in the Copenhagen area (Rigshospitalet, Herlev Hospital, and Hvidovre Hospital) in the period April 2016 to October 2018 were invited to participate [18, 19]. The three recruiting hospitals are public and serve a broad population with mixed socioeconomic backgrounds. The cardiac evaluation consisted of transthoracic echocardiography and ECG performed during the first month of life. Information on neonatal sex, gestational age (GA), age, weight, height, and body surface area (BSA; calculated by the formula suggested by Haycock [20]) at the time of postnatal cardiac examination were registered. Neonates that did not have echocardiography and ECG performed on the same day were excluded (*n* = 33) and a total of 17,450 neonates were identified for the study.

The study followed the Helsinki Declaration and was approved by the Regional Ethical Committee (H-16001518) and the Danish Data Protection Agency (I-suite 04546, HGH-2016–53). Written consent was obtained from parents.

### Electrocardiography

All ECGs were digitally recorded using the MAC 5500 HD system (GE ECG System, Milwaukee, USA), with a paper speed of 25 mm/sec, sensitivity of 10 mm/mV, sample rate of 500 Hz, bandwidth filter of 0.16–150 Hz, and stored in an ECG management system (MUSE, Version 8, GE Healthcare, Milwaukee, USA). The algorithm creates a median QRS complex for each lead and then determines the on- and offsets of the P, QRS, and T waves in a specific order. The ECGs were obtained when the neonates were relaxed or asleep and included lead I, II, III, aVR, aVL, aVF, V1, and (in most cases) V6 (*n* = 11,948). To ensure adequate data quality extensive manual validation was performed [21–23]. In this study, the following ECG features were analyzed: the QRS duration, the QRS area in V1 and V6, the absolute sum of the QRS areas in V1 and V6 (V1 + V6), the maximum S-wave amplitude in V1 (S-V1) and the maximum R-wave amplitude in V6 (R-V6), the sum of the maximum amplitudes in S-V1 and R-V6 (S-V1 + R-V6), and the Sokolow–Lyon voltage product defined as QRS_duration_·(S-V1 + R-V6) [24].

### Echocardiography

Two-dimensional transthoracic echocardiograms were performed with Vivid E9 ultrasound equipment according to a systematic protocol [18]. Standard sub-xiphoid, apical, left parasternal, and suprasternal views were acquired with 12 MHz and 6 MHz cardiac transducers. All projections and measurements were performed in accordance with the American Society of Echocardiography’s guidelines for pediatric echocardiography [25]. All raw data (cine loops and measurements) were acquired using EchoPac software version 113 (GE Healthcare, Horten, Norway). Cardiologists specialized in pediatric echocardiography reviewed all echocardiographic findings suspected to be abnormal. In the present study, a persistent ductus arteriosus or an interatrial communication were not included in the definition of CHD, due to the age spectrum in our cohort. The interventricular septal thickness at end-diastole (IVSd), left ventricular internal diameter at end-diastole (LVIDd), and left ventricular posterior wall thickness at end-diastole (LVPWd) were determined from the 2D parasternal long-axis view. LVM was calculated by the “cube” formula suggested by Devereux and Reichek [26]: 0.8{1.04 · ([IVSd + LVIDd + LVPWd]^3^ − LVIDd^3^)} + 0.6 which has been validated for use in children, and measured in grams [26]. In the present study, we defined neonates with LVMI above or equal to the 98th percentile as being LVMI outliers.

### Statistical Analyses

Baseline characteristics are presented as absolute values (percentages) for categorical data and continuous data are presented as median values (interquartile ranges; IQR). Neonates were divided into groups defined by postnatal age at the time of cardiac examination. Echocardiographic and ECG measurements are shown as median values with 2nd and 98th percentiles. Comparisons between groups were performed with Student’s *t*-test, or Wilcoxon Rank sum test, when appropriate. Linear regression analyses were used to investigate and illustrate the relationship between postnatal age, QRS features, and LVMI. To explore if outliers of the investigated ECG features (defined as ≥ 98th percentile) were useful as a diagnostic tool for identifying LVMI outliers, sensitivity and specificity analyses were performed. Furthermore, as a cut-off value defined as ≥ 98th percentile could be considered somewhat arbitrary, we also investigated the effect of other cut-off values (≥ 90th and ≥ 95th percentiles) and investigated the effect on sensitivities and specificities with these adjusted thresholds. Receiver operator characteristics curves (ROC) were constructed, areas under the curves (AUC) were calculated for each ECG feature and the findings were compared. To assess the measurement uncertainty binomial 95% confidence intervals (95% CI) were shown for sensitivity, specificity, and AUC values. R statistical software v. 1.4.1717 (Boston, MA, USA) was used for statistical analyses. A *p*-value < 0.05 was considered significant.

## Results

### Study Population

A total of 17,450 neonates were included (Table 1) with an approximately equal sex distribution (52% boys). The median postnatal age at examination was 11 days (range 0–30 days), GA was 281 days (range 212–301 days), weight was 3.6 kg (range 1.4–6 kg), height was 52 cm (range 38–68 cm), and BSA was 0.32 m^2^ (range 0.12–0.32 m^2^). The maternal ethnic composition was 92.3% Caucasian, 3.4% Asian, 1.5% Middle Eastern, 1% Black, and 1.8% other/mixed.

**Table 1:** Baseline characteristics of the cohort (*n* = 17,450)

Sex, boys	9020 (52%)
Postnatal age, days	11 (7–14)
Heart rate, bpm	143 (128–158)
Gestational age, days	281 (274–287)
Weight, kg	3.6 (3.3–4.0)
Height, cm	52 (51–54)
Body surface area, m^2^	0.23 (0.22–0.25)
Maternal caucasian ethnicity^a^	14,465 (92%)

Continuous variables are displayed as medians (interquartile ranges) and categorical variables as absolute values (percentages)

*bpm* beats per minute

^a^Data available in 15,712 mothers

### Postnatal Development in ECG Features

We found a poor correlation between ECG parameters and postnatal age, with *R*^2^ values ranging from 0.0002 to 0.0114 in linear regression analyses.

#### QRS Duration

**Fig. 1:**
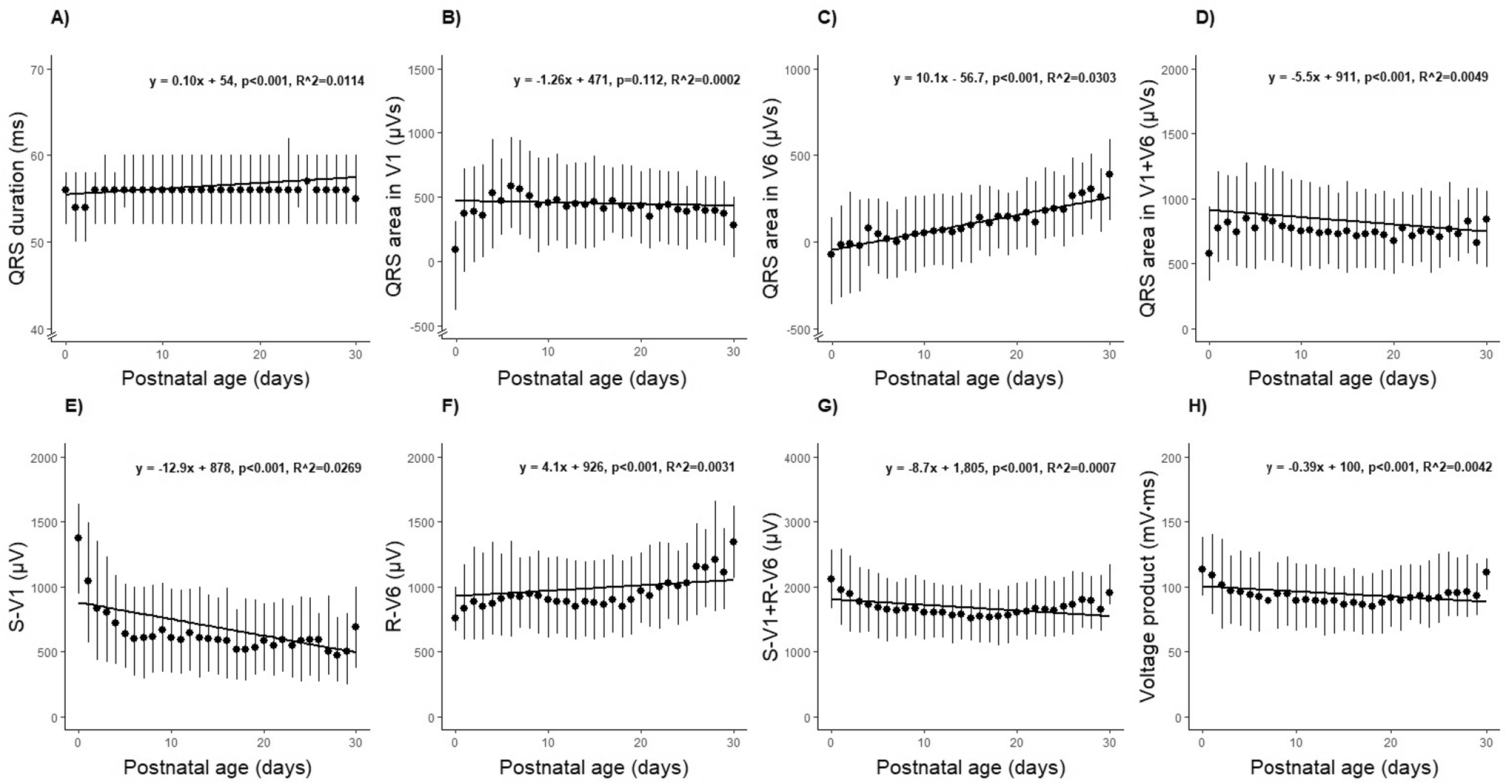
QRS complex features as a function of postnatal age (days). **A** QRS duration. **B** QRS area in V1. **C** QRS area in V6. **D** Absolute sum of the QRS area in V1 and V6 (V1 + V6). **E** Maximum S-V1 amplitude. **F** Maximum R-V6 amplitude. **G** Sum of maximum S-V1 and R-V6 amplitudes (S-V1 + R-V6). **H** Voltage product. Dots denote median values and vertical lines denote 25–75 percentiles. Solid horizontal line denotes linear regression

The median QRS duration was 56 ms (range 30–94 ms), which slightly increased during the first month of life; 54 ms (range 32–76 ms) at ages 0–4 days increasing to 56 ms (range 30–74 ms) at ages 25–30 days (*p* < 0.001; Fig. 1A and Online Resource 1). Boys had a significantly longer median QRS duration than girls (56 vs. 54 ms, *p* < 0.001).

#### Precordial QRS Areas, Amplitudes, and Voltage Product

The median sum of the QRS area in V1, V6, and their absolute sum was 445 µVs (range − 2693–3436 µVs), 74 µVs (range − 2121–3648 µVs), and 760 µVs (range 11–5592 µVs), respectively (Fig. 1 and Online Resource 1). The QRS area in V6, and the sum of the QRS areas in V1 and V6 increased significantly (both *p* < 0.05) during the first month of life, while the QRS area in V1 did not change significantly (*p* = 0.48). The median values of the maximum amplitudes of S-V1, R-V6, and S-V1 + R-V6 were 625 µV (range 19–3935 µV), 903 µV (range 24–3574 µV), and 1635 µV (range 131–5423 µV), respectively, and all three parameters changed significantly during the first month of life (all *p* < 0.05; Fig. 1 and Online Resource 1). The median voltage product was 91.6 mV ms (range 6.8–305.0 mV ms) which decreased during the first month of life (*p* < 0.01; Fig. 1 and Online Resource 1).

### Echocardiographic Findings

**Fig. 2:**
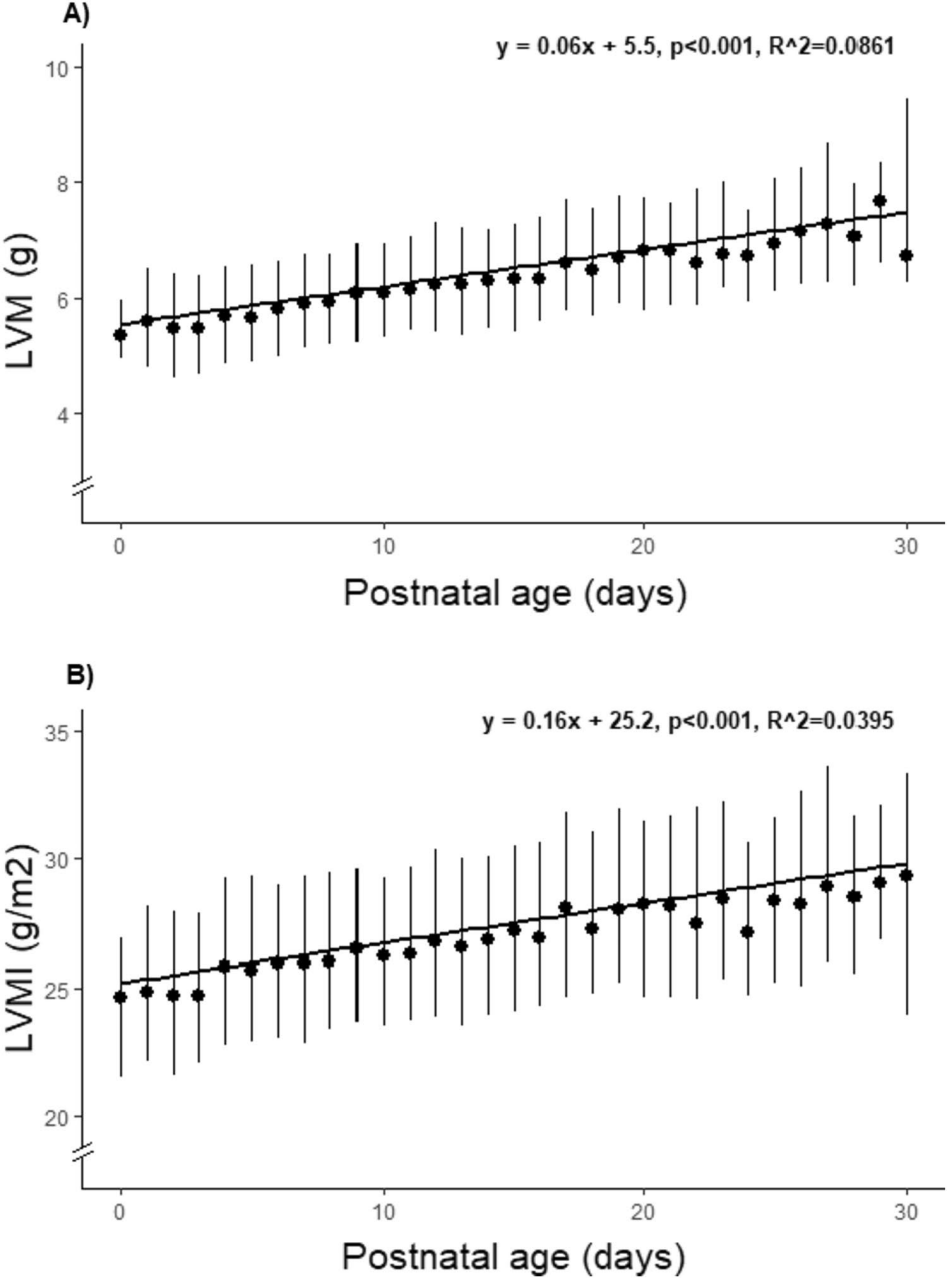
**A** Left ventricular mass (LVM) as a function of postnatal age at examination. **B** Left ventricular mass index (LVMI) as a function of postnatal age at examination. Dots denote median values and vertical lines denote 25–75 percentiles. Solid horizontal line denotes linear regression

Neonates with echocardiographic abnormalities were not excluded (~ 4% of the cohort), and the most common echocardiographic findings were minor abnormalities such as a small ventricular septal defect (VSD), bicuspid aortic valve, mitral valve disease, and pulmonary stenosis. None of the neonates were diagnosed with cardiac diseases associated with severe neonatal LVH, such as Noonan’s syndrome, Danon’s or Pompe’s diseases. The median IVSd was 2.5 mm (range 0.8–5.1 mm), LVIDd was 20.0 mm (range 11.6–28.0 mm), and LVPWd was 2.0 mm (range 0.7–5.7 mm). The median LVM was 6.1 g (range 2.4–14.0 g) and LVMI was 26.5 g/m^2^ (range 11.1–60.2 g/m^2^). Analyzing LVM and LVMI as a function of age we found a significant increase during the first month of life (5.5 vs. 7.1 g and 24.7 vs. 28.6 g/m^2^; 0–4 days vs. 25–30 days; both *p* < 0.001, Fig. 2 and Online Resource 1). Boys had higher LVM and LVMI than girls (6.3 vs. 6.0 g and 26.9 vs. 26.1 g/m^2^, respectively; both *p* < 0.001).

### Sensitivity and Specificity of ECG Features for Identifying LVMI Outliers

We found no or low correlation between LVMI and the investigated ECG features (Table 2 and Online Resource 3). To further investigate if outlying ECG features (≥ 98th percentile) could be used to identify LVMI outliers in neonates, sensitivity and specificity analyses were performed (Table 2 and Online Resource 2). Sensitivities for all the investigated ECG features were low, ranging from 0% [0–1.9 95% CI] to 9.0% [6.0–12.7 95% CI], with the highest value for the QRS duration (9.0% [6.0–12.7 95% CI]; Table 2). Specificities were generally high, ranging from 97.2% [97.0–97.4 95% CI] to 98.0% [97.8–98.3 95% CI]. The sensitivity and specificity did not improve considerably when stratifying neonates by postnatal age at examination (Table 2). After adjustment of the defined ECG features’ thresholds (≥ 90th, ≥ 95th, and ≥ 98th percentiles) we observed a small increase in sensitivities, with the highest value for the QRS duration (21.7%; QRS duration ≥ 90th percentile, and LVMI ≥ 98th percentile). The specificities were generally still high, ranging from 89.1 to 98.1%, after adjustment. However, no ECG feature showed a strong discriminatory power for identifying LVMI outliers after these adjustments (Online Resource 2).

**Table 2:** Sensitivity, specificity, and AUC (95% CI) analyses of ECG features for identifying LVMI outliers stratified by postnatal age at examination

	All (*n* = 17,450)	0–9 days (*n* = 6659)	10–19 days (*n* = 8708)	20–30 days (*n* = 2083)	AUC for ECG features
QRS duration ≥ 98 percentile	9.0 (6.0–12.7)	5.7 (2.3–11.4)	11.2 (6.8–17.1)	7.9 (1.7–21.4)	0.61 (0.57–0.64)
	97.2 (97.0–97.4)	95.9 (95.4–96.4)	96.7 (99.6–99.7)	95.5 (94.5–96.4)	
QRS area in V1 ≥ 98 percentile	3.1 (1.4–8.8)	1.8 (0.22–6.4)	4.2 (1.5–8.9)	6.5 (0.79–21.4)	0.53 (0.50–0.56)
	98.0 (97.7–98.2)	97.9 (97.5–98.3)	98.1 (97.8–98.4)	98.0 (97.2–98.6)	
QRS area in V6 ≥ 98 percentile	1.5 (0.3–4.4)	0 (0–5.3)	1.9 (0.23–6.6)	4.2 (0.11–21.1)	0.51 (0.47–0.55)
	98.0 (97.7–98.3)	97.9 (97.4–98.4)	98.0 (97.6–98.4)	97.9 (97.1–98.6)	
QRS area in V1 + V6 ≥ 98 percentile	2.7 (0.88–6.1)	1.6 (0.04–8.4)	5.1 (1.7–11.4)	0 (0–15.4)	0.55 (0.51–0.59)
	98.0 (97.8–98.3)	98.0 (97.4–98.4)	98.1 (97.8–98.5)	98.0 (97.1–98.7)	
S-V1 ≥ 98 percentile	2.4 (0.99–4.9)	2.7 (0.56–7.7)	2.8 (0.76–7.0)	3.2 (0.1–16.7)	0.55 (0.55–0.59)
	98.0 (97.8–98.2)	97.9 (97.5–98.3)	98.1(97.7–98.4)	97.9 (97.1–98.5)	
R-V6 ≥ 98 percentile	0 (0–1.9)	0 (0–5.3)	0 (0–3.4)	0 (0–14.3)	0.51 (0.47–0.55)
	98.0 (97.7–98.2)	97.8 (97.3–98.3)	97.9 (97.5–98.3)	97.9 (97.1–98.6)	
S-V1 + R-V6 ≥ 98 percentile	2.7 (0.9–6.1)	1.6 (0.04–8.4)	2.0 (0.25–7.1)	9.1 (1.1–29.2)	0.52 (0.48–0.57)
	98.0 (97.8–98.3)	98.0 (97.5–98.4)	98.0 (97.6–98.4)	98.0 (97.1–98.7)	
Voltage product ≥ 98 percentile^a^	2.7 (0.9–6.1)	4.7 (1.0–13.1)	2.0 (0.25–7.1)	9.1 (1.1–29.2)	0.49 (0.45–0.54)
	98.0 (97.8–98.3)	98.0 (97.6–98.5)	98.0 (97.6–98.4)	98.1 (97.2–98.8)	

Data are displayed as sensitivity (95% confidence interval; top row in each cell), and specificity (95% confidence interval; bottom row in each cell) in percentages. LVMI (left ventricular mass index) outliers defined as LVMI ≥ 98 percentile

*AUC* area under the curve, *S-V1* maximum S-wave amplitude in V1, *R-V6* maximum R-wave amplitude in V6

^**a**^Sokolow–Lyon voltage product: QRS_duration_·(S-V1 + R-V6)

**Fig. 3:**
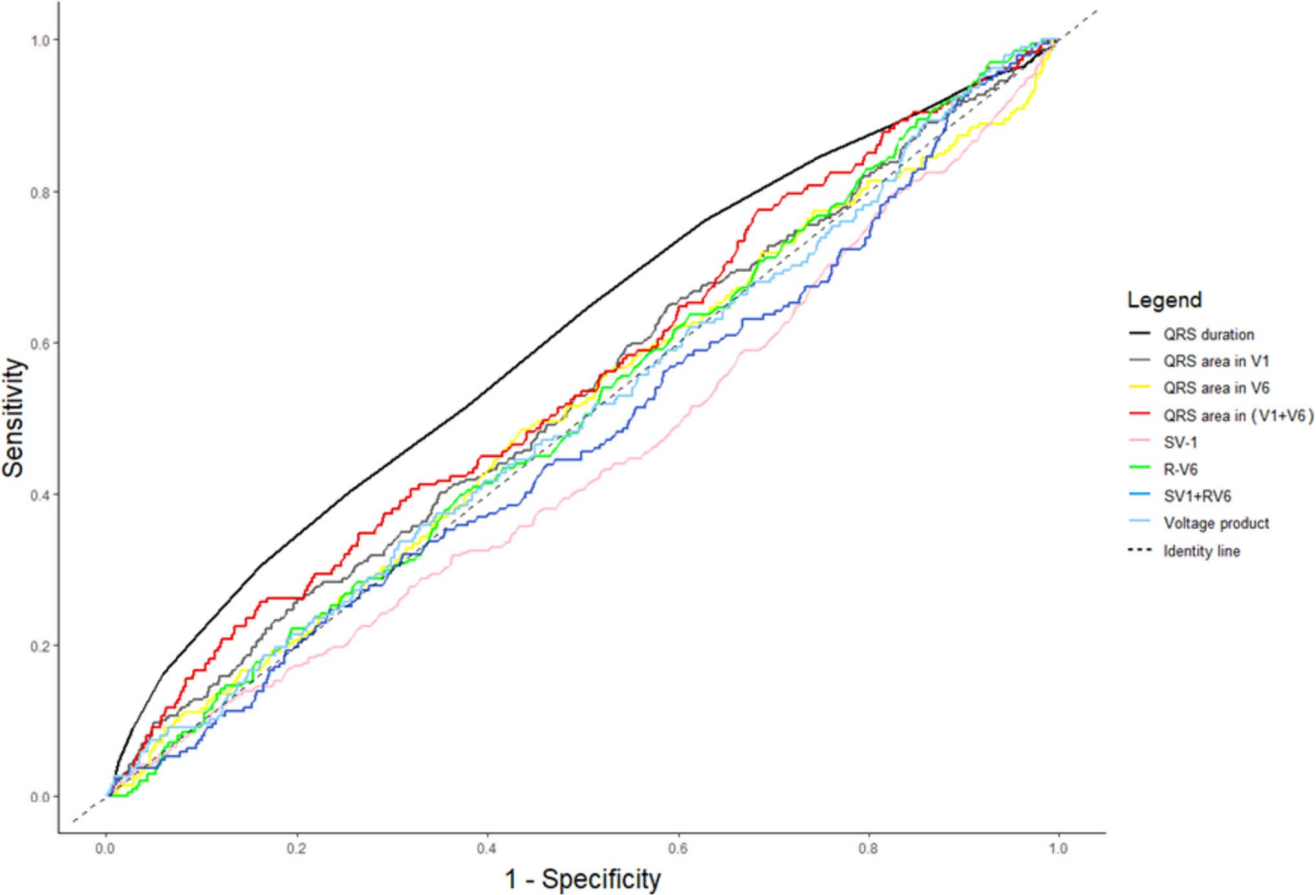
ROC curve for each ECG feature (1–100 percentile) to identify left ventricular mass index (LVMI) outliers (LVMI ≥ 98 percentile). The area under the ROC curve (AUC) denotes the ability of the ECG feature as a screening tool to identify LVMI outliers. All lines are close to the identity line (AUC = 0.50) indicating poor performance of the ECG features as a diagnostic tool for identifying LVMI outliers. *S-V1* maximum S-wave amplitude in V1, *R-V6* maximum R-wave amplitude in V6. Sokolow–Lyon voltage product: QRS_duration_·(S-V1 + R-V6)

ROC and AUC analyses for each ECG feature showed AUC values between 0.49 [0.45–0.54 95% CI] and 0.61 [0.57–0.64 95% CI] (Table 2 and Fig. 3); all close to the identity line (AUC = 0.50). When comparing AUC for each ECG feature, we found the highest value for the QRS duration (0.61 [0.57–0.64 95% CI]) and the lowest value for the voltage product (0.49 [0.45–0.54 95% CI]).

## Discussion

In the present large, population-based cohort study of 17,450 neonates, we assessed QRS complex features and LVMI during the first month of life. Updated standard reference values for both electro- and echocardiographic parameters are presented. Our study showed that most investigated QRS features evolved during the first month of life. However, we found no to low correlation between QRS features and LVMI resulting in low sensitivity, but high specificity of these parameters to identify LVMI outliers in neonates.

There are a limited number of published smaller, reference studies [27–30] (*n* = 44–668) investigating QRS features in neonates. Rijnbeek et al. [27] (*n* = 44) investigated the QRS duration in neonates and found a median value of 67 ms, while Davignon et al. [30] (*n* = 668) found a value of ~ 50 ms in V5; relatively similar to our findings of 56 ms. Saarel et al. [31] (*n* = 257) found a median QRS duration of 60 ms for boys and 58 ms for girls; quite similar to our findings of 56 ms for boys and 54 ms for girls. Investigating precordial amplitudes during the first month of life, Davignon et al. reported a decrease in S-V1 (from ~ 800 to ~ 400 µV) very similar to our findings (from 825 to 517 µV), as well as an increase in R-V6 (from ~ 370 to  ~ 700 µV). We also found an increase in R-V6, but the absolute values differed to some extent (from 859 to 1113 µV). Investigating the influence of sex on R-V6 in neonates, Saarel et al. found no effect of sex and median values of 665 µV for boys and 773 µV for girls. We have also previously documented no effect of sex on R-V6, as well as a large variation in absolute values for neonatal precordial amplitudes [23]. Furthermore, these differences most likely reflect variation in applied methodologies, including use of manual vs. automated measurements, one lead vs. all leads, differences in neonatal characteristics and cohort sizes, etc. Taken together, the current study is the first to provide thorough reference values for a wide range of neonatal QRS features during the first month of life.

Previous pediatric studies [9, 15, 17, 32–34] (*n* = 12–3209) investigating the association between LVM/LVMI and QRS features have produced mixed results, and only few of these studies have focused on neonates. One of our main findings is no—to low—correlation between LVMI and QRS features resulting in low sensitivities (range 0–9%), generally high specificities (range 97.2–98.0%), and AUC values close to the identity line (range 0.494–0.607). Comparable to our results, Rivenes et al. [15] (*n* = 1688; 0–14 years) found a low sensitivity (< 20%), but a high specificity (range 76–99%) for a range of ECG criteria for detecting echocardiographic LVH, regardless of HIV status which was the study’s main aim. Similarly, Rijnbeek et al. [9] (*n* = 832; age 0–15 years) found low sensitivity (< 25%) for ECG criteria to identify high LVMI and reported that factors such as the applied LVH definitions, combination of several ECG criteria, and consideration of clinical indexes of volume and/or pressure overload, affected sensitivity. Contrary, Tauge et al. [17] (*n* = 3209; age 0–18 years) found a high sensitivity (≥ 90%), and a low specificity (43%); however this cohort had a very high prevalence of ECG-LVH, but low prevalence of echocardiographic-LVH, likely explaining the findings. Overall, limitations of the mentioned studies include the often small sample sizes, large variation in the age of the included children, and heterogenous cohort compositions (including children with CHD and/or other comorbidities). In our study, we did not find a noticeable effect on sensitivity after adjustment of the definition of LVMI outliers, but adjustment of the thresholds for QRS features had a minor effect.

Based on data from 17,450 unselected neonates from the general population, our study is to date the largest study presenting reference values for QRS complex features and their association with LVMI. Our findings show that QRS features are not reliable predictors of LVMI, consistent with most previous studies. However, we investigated LVMI outliers, as defined by ≥ 98th percentile, but all these neonates may not have true pathological left ventricular hypertrophy and greater sensitivity for ECG features in identifying LVH has been reported in smaller, selected cohorts enriched for CHD [32, 33]. Sensitivity for children with VSDs (*n* = 12) has been found to be 60%, with aortic stenosis (*n* = 19) up to 67%, and up to 71% in children with rheumatic heart disease (*n* = 84). ECG is a non-invasive, cost-effective, and easily obtainable diagnostic tool and continues to have diagnostic significance in neonates presenting with symptoms consistent with arrhythmia, suspicion of genetic channelopathy, drug side effect, etc., but is an insensitive screening tool for identifying LVMI outliers in unselected/asymptomatic neonates.

There are limitations to the present study. The sensitivity and specificity of a given diagnostic modality is dependent on the prevalence of the condition it is used to diagnose, i.e. had we investigated selected groups of hospitalized neonates with, e.g. hypertrophic cardiomyopathy, congenital aortic stenosis, or another major CHD, the sensitivity and specificity of QRS complex features to diagnose LVMI would likely have been higher. The ECGs were recorded with eight leads instead of twelve due to logistical reasons and considerations of participant discomfort, some inter-observational variation in measurements cannot be ruled out [35], and external validation was not performed. Furthermore, ethnic differences may exist, and the results may not be generalizable to populations with different ethnic distributions. Previous studies have shown that non-ethnicity-specific LVH criteria result in overestimations for African-Americans and may underestimate LVH in white populations [36]. Lastly, the use of the “cube” formula to calculate LVM has certain prerequisites and may be vulnerable to measurement inaccuracies explaining some of the differences in absolute values seen when comparing to previous studies. Other formulas for LVM calculation, such as, e.g. the 5/6 AL method, may be more precise and should be investigated in future projects.

In conclusion, the study presents updated reference values for QRS complex features and their association with LVMI in neonates from a large, unselected, population-based cohort. The QRS complex gradually evolved during the first month of life but had low correlation with LVMI. ECG features were associated with low sensitivity, but high specificity, and are therefore not reliable indicators of LVMI outliers. Taken together, our results do not support the use of QRS features as a diagnostic tool for identifying LVMI outliers in asymptomatic neonates.

### Supplementary Information

Below is the link to the electronic supplementary material.

Supplementary file1 (DOCX 132 kb)

## Data Availability

Due to participant confidentiality the full data set underlying this article cannot be made shared publicly.

## Abbreviations

AUC: Area under the curve
BMI: Body mass index
BSA: Body surface area
CHD: Congenital heart disease
ECG: Electrocardiogram
GA: Gestational age
IVSd: Interventricular end-diastolic septal thickness
IQR: Interquartile ranges
HIV: Human immunodeficiency virus
LVH: Left ventricular hypertrophy
LVIDd: Left ventricular internal end-diastolic diameter
LVM: Left ventricular mass
LVMI: Left ventricular mass index
LVPWd: Left ventricular end-diastolic posterior wall thickness
ROC: Receiver operator characteristics curves
VSD: Ventricular septal defect
