# Supplementary material for: The Neonatal QRS Complex and Its Association with Left Ventricular Mass
Source: Pediatr Cardiol. 2023 Dec 27;45(2):248–56. doi: 10.1007/s00246-023-03361-0 (PMC10822000; doi:10.1007/s00246-023-03361-0)
Supplement: Supplementary file 1 — Supplementary file1 (DOCX 132 kb) [file 246_2023_3361_MOESM1_ESM.docx]

**SUPPLEMENTARY**

**THE NEONATAL QRS COMPLEX AND ITS ASSOCIATION WITH LEFT VENTRICULAR MASS**

Julie Molin^1^, MD; Joachim Hartmann^1^, MD; Maria Munk Pærregaard^1^, MD; Caroline Boye Thygesen^1^, MD; Anne-Sophie Sillesen^1^, MD, PhD; Anna Axelsson Raja ^2^, MD, PhD; Ruth Ottilia Birgitta Vøgg^1^, MD, PhD; Kasper Karmark Iversen^1,3^, MD, DMSc; Henning Bundgaard^2,3^, MD, DMSc; Alex Hørby Christensen^1,2,3*^, MD, PhD.

**Affiliations:**

^1^Department of Cardiology, Herlev-Gentofte Hospital, Copenhagen University Hospital, Copenhagen, Denmark.

^2^The Capital Regions Unit for Inherited Cardiac Diseases, Department of Cardiology, The Heart Center, Rigshospitalet, Copenhagen University Hospital, Copenhagen, Denmark.

^3^Department of Clinical Medicine, University of Copenhagen, Copenhagen, Denmark.

**^*^Corresponding Author:**

Alex Hørby Christensen, Consultant, PhD, Associate professor

Department of Cardiology, Copenhagen University Hospital - Herlev-Gentofte Hospital, Borgmester Ib Juuls Vej 1, DK-2730 Herlev, Denmark, e-mail: [alexhc@dadlnet.dk](mailto:alexhc@dadlnet.dk); Phone: +45 50517681

**Statements and Declarations:**

**Conflict of interest:** The authors have no conflicts of interest relevant to this article to disclose. The authors have no financial relationships relevant to this article to disclose.

| **Online Resource 1. Electrocardiographic and echocardiographic parameters stratified by postnatal age at examination (n=17,450)** | | | | | | | | |
| --- | --- | --- | --- | --- | --- | --- | --- | --- |
|  | **All**  **(n=17,450)** | **0**–**4 days**  **(n=2,740)** | **5**–**9 days (n=3,919)** | **10**–**14 days (n=6,486)** | **15**–**19 days (n=2,222)** | **20**–**24 days (n=1,258)** | **25**–**30 days (n=825)** | **P-value*** |
| **QRS duration, ms** | 56  (44–68) | 54  (40–66) | 56  (44–68) | 56  (44–68) | 56  (44–69) | 56  (44–68) | 56  (42–68) | **<0.001** |
| **QRS area in V1,** **µVs** | 445  (-623–1,598) | 397  (-872–1,671) | 503  (-545–1,710) | 447  (-625–1,602) | 446  (-521–1,470) | 427  (-493–1,359) | 396  (-424–1,286) | 0.48 |
| **QRS area in V6, µVs** | 74  (-745–898) | -4  (-899–870) | 29  (-773–877) | 64  (-730–885) | 122  (-598–870) | 160  (-563–904) | 252  (-470–1,054) | **<0.001** |
| **QRS area in (V1+V6), µVs** | 760  (120–2,128) | 798  (139–2,303) | 798  (120–2,224) | 745  (120–2,133) | 733  (123–1,905) | 730  (82–1,870) | 757  (139–1,909) | **0.03** |
| **S**–**V1, µV** | 625  (83–2,070) | 825  (83–2,543) | 634  (83–1,970) | 610  (87–1,933) | 561  (73–1,807) | 566  (73–1,711) | 517  (82–1,659) | **<0.001** |
| **R**–**V6, µV** | 903  (239–2,133) | 859  (214–2,163) | 927  (258–2,119) | 878  (214–2,072) | 883  (258–2,001) | 971  (312–2,227) | 1,113  (366–2,348) | **<0.001** |
| **S**–**V1+R**–**V6, µV** | 1,635  (580–3,284) | 1,849  (583–3,778) | 1,664  (594–3,314) | 1,586  (565–3,115) | 1,523  (556–3,065) | 1,634  (620–2,983) | 1,752  (661–3,168) | **<0.001** |
| **Voltage product, mV•ms^†^** | 91.6  (31.6–186.1) | 101.2  (31.1–212.9) | 93.1  (32.0–187.7) | 89.3  (30.6–177.8) | 86.2  (30.5–171.6) | 91.6  (33.7–170.5) | 95.3  (39.0–182.6) | **<0.01** |
| **LVM, g** | 6.1  (3.8–9.7) | 5.5  (3.7–8.8) | 5.9  (3.8–9.2) | 6.2  (4.0–9.5) | 6.5  (4.2–10.0) | 6.7  (4.2–10.2) | 7.1  (4.2–11.0) | **<0.001** |
| **LVMI, g/m^2^** | 26.5  (17.8–39.0) | 24.7  (16.0–36.3) | 26.0  (17.6–38.1) | 26.6  (18.5–38.6) | 27.6  (18.9–40.1) | 27.9  (18.8–40.0) | 28.6  (18.8–43.0) | **<0.001** |
| Data are displayed as medians (2-98%iles). Abbreviations: S-V1: maximum S-wave amplitude in V1; R-V6: maximum R-wave amplitude in V6. *Comparison between neonates in age group 0-4 days and 25-30 days. **^†^**Sokolow-Lyon voltage product: QRS_duration_•(S-V1+R-V6). Significant values are marked with **bold**. | | | | | | | | |

| **Online Resource 2. Sensitivity and specificity analyses of ECG features for identifying LVMI outliers.** | | | |
| --- | --- | --- | --- |
|  | **LVMI**≥**90%ile** | **LVMI**≥**95%ile** | **LVMI**≥**98%ile** |
| **QRS duration**≥**90%ile** | 17.5 (90.7) | 18.9 (90.3) | 21.7 (90.1) |
| **QRS duration**≥**95%ile** | 11.3 (94.5) | 12.8 (94.3) | 16.1 (94.1) |
| **QRS duration**≥**98%ile** | 5.6 (97.3) | 6.3 (97.2) | 9.0 (97.2) |
| **QRS area in V1**≥**90%ile** | 11.9 (90.1) | 10.9 (89.9) | 12.8 (89.9) |
| **QRS area in V1**≥**95%ile** | 6.9 (95.2) | 7.2 (95.1) | 9.3 (95.1) |
| **QRS area in V1**≥**98%ile** | 2.6 (98.0) | 2.6 (98.0) | 3.1 (98.0) |
| **QRS area in V6>90%ile** | 9.2 (90.0) | 10.2 (90.1) | 11.1 (90.1) |
| **QRS area in V6>95%ile** | 4.8 (95.0) | 5.2 (95.1) | 6.1 (95.1) |
| **QRS area in V6**≥**98%ile** | 2.2 (98.0) | 2.5 (98.0) | 1.5 (98.0) |
| **QRS area in (V1+V6)**≥**90%ile** | 13.1 (90.3) | 13.7 (90.2) | 16.6 (90.1) |
| **QRS area in (V1+V6)**≥**95%ile** | 6.8 (95.2) | 7.2 (95.1) | 9.1 (95.1) |
| **QRS area in (V1+V6)**≥**98%ile** | 2.3 (98.1) | 2.6 (98.1) | 2.7 (98.0) |
| **S-V1**≥**90%ile** | 7.7 (89.6) | 6.7 (89.7) | 9.7 (89.9) |
| **S-V1**≥**95%ile** | 3.9 (94.8) | 3.2 (94.9) | 4.5 (95.0) |
| **S-V1**≥**98%ile** | 1.9 (98.0) | 1.5 (98.0) | 2.4 (98.0) |
| **R-V6**≥**90%ile** | 11.9 (90.1) | 11.8 (89.9) | 9.6 (89.8) |
| **R-V6**≥**95%ile** | 6.2 (95.0) | 5.6 (94.9) | 3.0 (95.0) |
| **R-V6**≥**98%ile** | 2.2 (98.0) | 1.3 (98.0) | 0 (98.0) |
| **S-V1+R-V6**≥**90%ile** | 9.1 (89.9) | 8.4 (89.9) | 8.0 (89.9) |
| **S-V1+R-V6**≥**95%ile** | 4.7 (95.0) | 4.4 (95.0) | 3.7 (95.0) |
| **S-V1+R-V6**≥**98%ile** | 1.9 (98.0) | 2.2 (98.0) | 2.7 (98.0) |
| **Voltage product**≥**90%ile** | 10.8 (90.0) | 10.0 (90.0) | 9.6 (89.9) |
| **Voltage product**≥**95%ile** | 6.0 (95.1) | 6.4 (95.0) | 7.5 (95.0) |
| **Voltage product**≥**98%ile** | 2.2 (98.1) | 2.4 (98.1) | 2.7 (98.0) |
| Data are displayed as sensitivity (specificity) in percentages. LVMI (left ventricular mass index) outliers defined as LVMI≥90, ≥95, and ≥98%iles. Abbreviations: S-V1: maximum S-wave amplitude in V1; R-V6: maximum R-wave amplitude in V6. | | | |

**ONLINE RESOURCE 3**

**
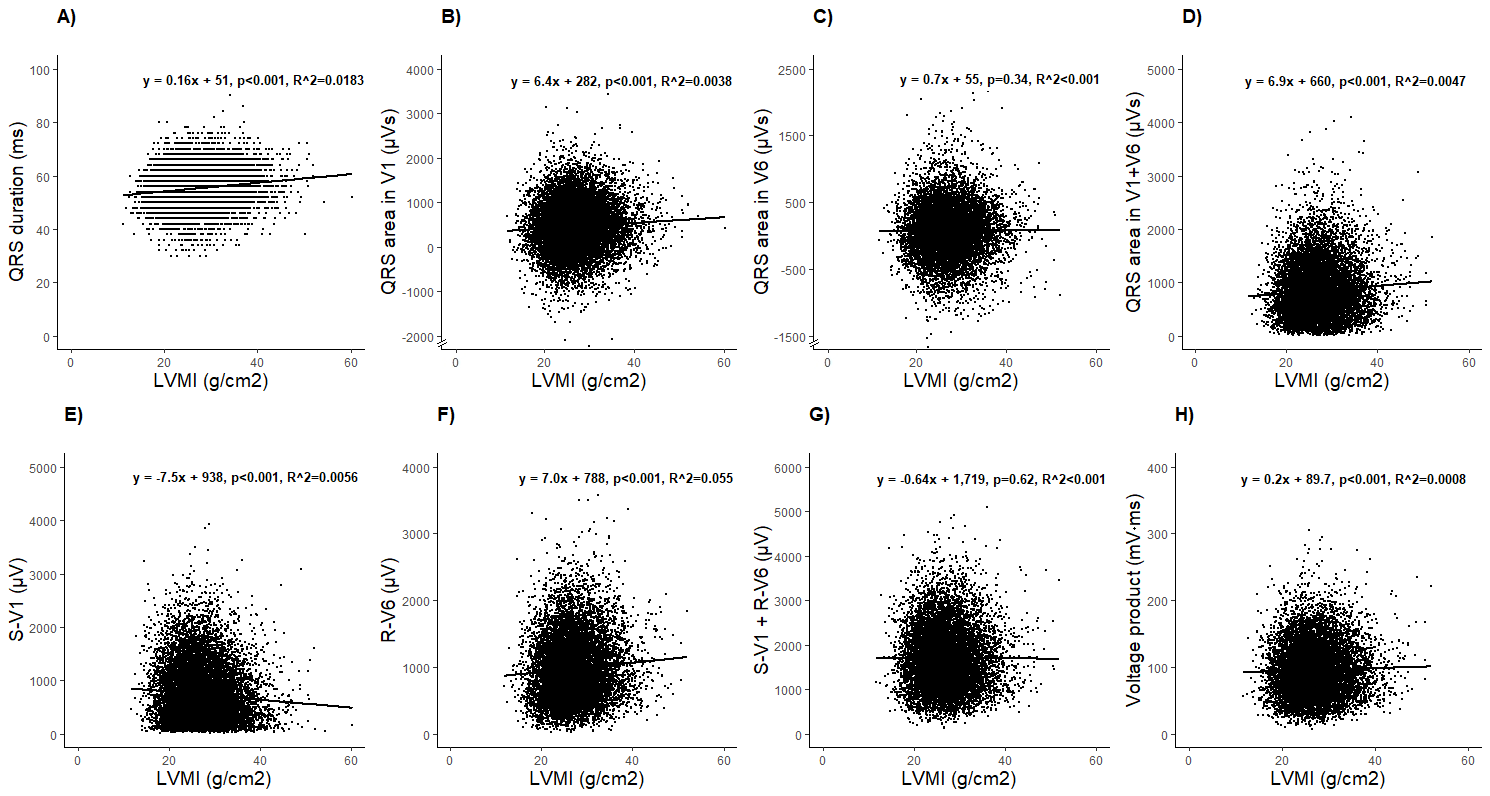
**

**FIGURE LEGENDS SUPPLEMENTARY**

**Online Resource** **3.** QRS complex features as a function of left ventricular mass index (LVMI). A) QRS duration; B) QRS area in V1; C) QRS area in V6; D) Absolute sum of QRS area in (V1+V6); E) Maximum S-V1 amplitude; F); Maximum R-V6 amplitude**;** G) Sum of maximum S-V1+R-V6 amplitudes; H) Voltage product.
